# A standardized surgical approach to appendiceal orifice tumors: a case report

**DOI:** 10.1093/jscr/rjaf259

**Published:** 2025-04-30

**Authors:** Banks Ford Harris, Camron Noah Collins, Justin D Gerard

**Affiliations:** College of Medicine, University of Arkansas for Medical Sciences, 4301 West Markham Street #550, Little Rock, AR 72205, United States; College of Medicine, University of Arkansas for Medical Sciences, 4301 West Markham Street #550, Little Rock, AR 72205, United States; Department of Surgery, Graduate School of Medicine, University of Tennessee Knoxville, 1924 Alcoa Highway, Box U-11, Knoxville, TN 37920, United States

**Keywords:** appendiceal orifice tumor, radical appendectomy with cecal cuff, CELS, sessile serrated lesion, surgical oncology, minimally invasive surgery

## Abstract

The optimal management of appendiceal orifice (AO) tumors remains a topic of debate due to their anatomical complexity and the absence of a standardized surgical approach. This case report describes the successful treatment of an incompletely resected sessile serrated lesion at the AO in a 47-year-old female using a multidisciplinary strategy. Preoperative imaging, gastroenterology consultation, and intraoperative specimen evaluation guided a laparoscopic appendectomy with cecal cuff resection, achieving complete tumor removal with minimal morbidity. This approach preserved ileocecal valve function and ensured accurate pathological assessment. This case highlights key considerations, including the importance of avoiding ileocecal valve compromise and ensuring polyp retrieval for definitive pathological assessment. We propose appendectomy with cecal cuff resection as a standardized, effective technique to reduce treatment variability and mitigate incomplete resection risks in AO tumors.

## Introduction

Appendiceal orifice (AO) tumors, particularly sessile serrated lesions (SSLs), present unique surgical challenges due to their location and potential for malignant transformation. The absence of a universally accepted resection strategy fuels ongoing debate. Historically, right hemicolectomy has been considered the gold standard for malignant AO tumors, offering comprehensive lymph node dissection and oncologic clearance but at the cost of significant morbidity and prolonged recovery [1]. For nonmalignant lesions, this approach often proves excessive. Alternatives, such as extended appendectomy with partial cecectomy or combined endoscopic-laparoscopic surgery (CELS), aim to balance oncologic safety with reduced invasiveness but carry distinct risks, including staple line complications, incomplete resection, or perforation [2, 3].

This report advocates laparoscopic appendectomy with cecal cuff resection as an optimal, organ-preserving solution for non-malignant AO tumors. By avoiding ileocecal anastomosis and preserving ileocecal function, this technique minimizes operative risks while ensuring complete tumor excision [4]. This case report highlights the value of multidisciplinary collaboration and proposes a standardized approach to enhance patient outcomes and reduce treatment inconsistencies.

## Case report

**Figure 1:**
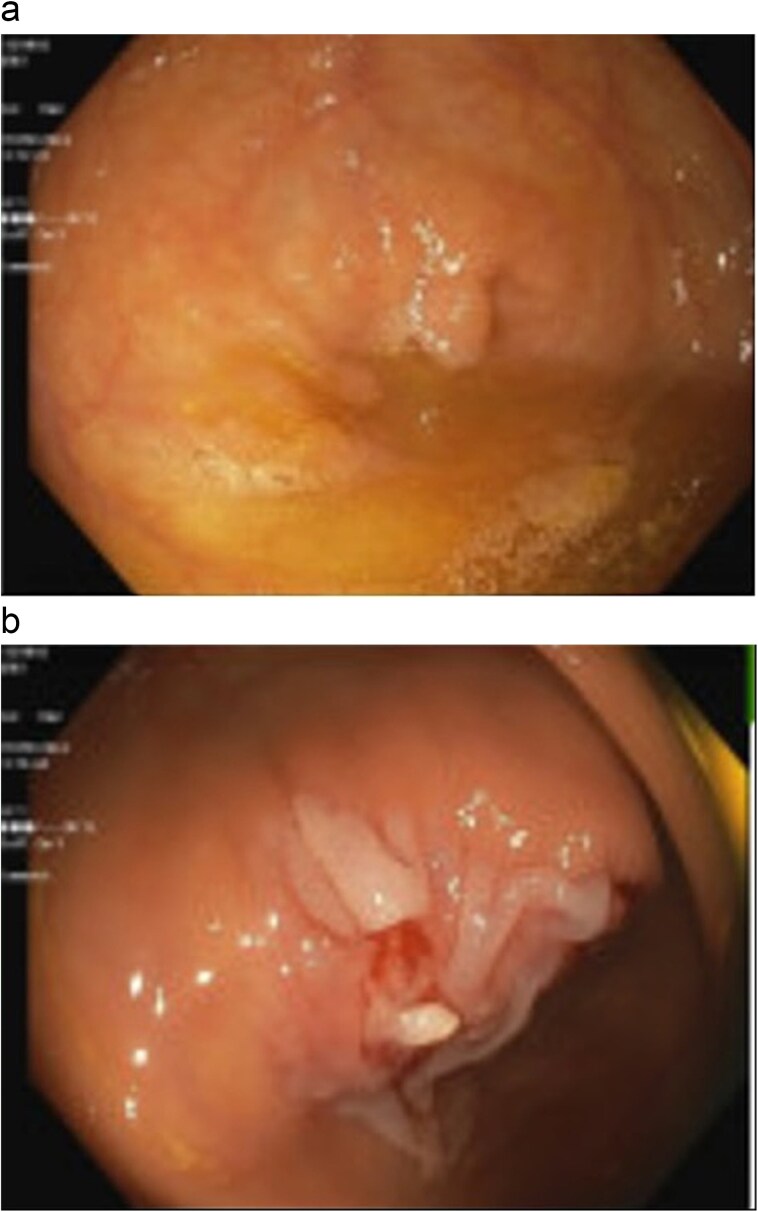
Appendiceal orifice with polyp (a); post-polypectomy incomplete resection (b).

A 47-year-old female with a past medical history of gastroesophageal reflux disease and hypothyroidism and a surgical history notable for a prior cesarean section, underwent her first screening colonoscopy. The procedure revealed an 8 mm sessile polyp at the appendiceal orifice (Fig. 1a). An initial attempt at cold snare polypectomy resulted in only partial resection (Fig. 1b). Histopathology confirmed an SSL without dysplasia. Given the incomplete resection and risk of potential malignant transformation, surgical consultation was obtained for definitive management.

Multidisciplinary preoperative planning with gastroenterology and radiology included a detailed review of the prior colonoscopy and CT imaging, confirming the lesion’s proximity to the AO. Although no mass was visualized on CT, the measured distance of 3.3 cm from the ileocecal valve to the base of the appendix provided confidence that a resection could be performed without compromising the valve. Given the concern about compromising and/or occluding the AO via endoscopic methods, a laparoscopic appendectomy with cecal cuff was selected as a targeted, organ preserving approach.

**Figure 2:**
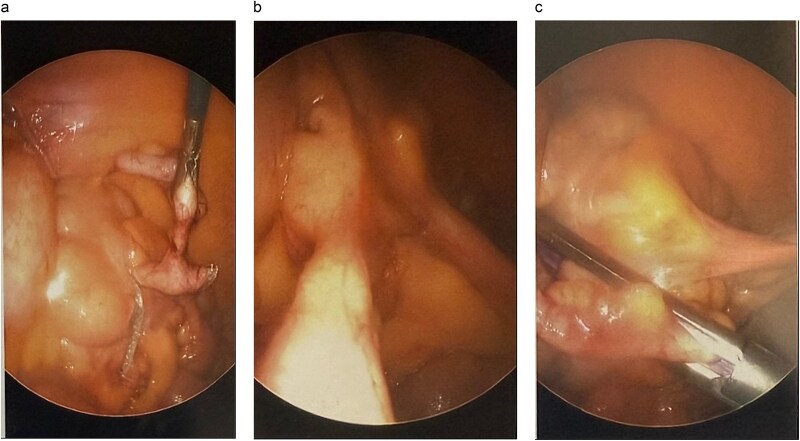
Intraoperative view of appendectomy with cecal cuff.

**Figure 3:**
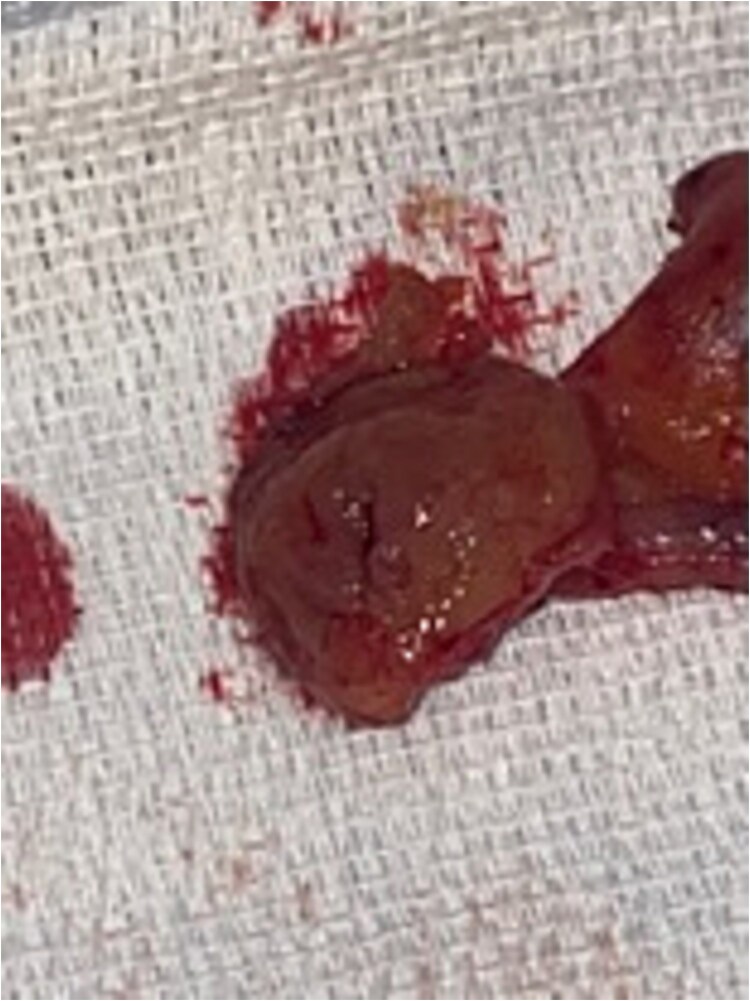
Resected appendix with cecal cuff and polyp.

The procedure employed standard laparoscopic appendectomy port placement. The appendix, appearing grossly normal, was mobilized, and the mesoappendix was divided using a LigaSure. Two purple EndoGIA stapler loads transected the appendix with a small cecal cuff, carefully sparing the ileocecal valve (Fig. 2) The specimen, retrieved via an Endo Catch bag, was inspected on the back table confirming complete resection of the polyp with grossly negative margins (Fig. 3). Hemostasis was confirmed, and the procedure was completed without complications. The patient had an uneventful recovery and was discharged following the operation.

## Discussion

The optimal surgical management of AO tumors remains a topic of debate due to varying oncologic risks and anatomic constraints [5, 6]. Right hemicolectomy, while offering the most oncologically complete resection, entails increased risk for morbidity such as anastomotic leak, postoperative ileus, and prolonged hospital stay [7]. Right hemicolectomy should be reserved for cases involving malignant transformation, high-risk histologic features, inability to completely resect the polyp with partial cecectomy, or concern about impingement upon the ileocecal valve with attempted partial resection. In cases of nonmalignant AO tumors, as demonstrated in this report, such an approach is often unnecessary and excessive. Extended appendectomy with partial cecectomy offers a less invasive alternative [2].

CELS, a minimally invasive hybrid technique, combines endoscopic polypectomy with laparoscopic oversight. While CELS has demonstrated effectiveness in managing some AO polyps, it presents unique risks. The thin-walled nature of the cecum predisposes it to an increased risk of perforation with CELS, and manipulation near the AO can induce periappendiceal edema and subsequent occlusion, which may precipitate appendicitis [8, 9]. Moreover, CELS demands precise interdisciplinary coordination and may fail to ensure complete resection in anatomically challenging cases [10].

We propose laparoscopic appendectomy with a cecal cuff as a safer and more definitive surgical approach for managing AO tumors. By excising a minimal cecal margin alongside the appendix, this approach enables resection without an anastomosis, preserving cecal integrity and bowel function. To optimize outcomes, the patient should be positioned in lithotomy, with gastroenterology on call for attempted CELS procedure if safe resection of the polyp or cecal cuff risks compromising the ileocecal valve. Intraoperative specimen assessment confirms complete excision, reducing the risk of missed polyp resection. Compared to CELS, which carries higher rates of bleeding and secondary interventions, this technique may offer lower complication rates and greater reliability, particularly for AO-involved lesions [11].

## Conclusion

Managing AO tumors demands a tailored, yet standardized, approach that balances oncologic efficacy with patient safety. We propose a hierarchy of interventions: laparoscopic appendectomy with cecal cuff as the preferred initial strategy, followed by CELS, then right hemicolectomy. Laparoscopic appendectomy with cecal cuff optimizes outcomes by ensuring complete resection, minimizing complications, and avoiding unnecessary colectomies. Adoption of this approach as a standard could streamline care and improve consistency in AO tumor management.
